# Targeting Metabolic Syndrome in Hidradenitis Suppurativa by Phytochemicals as a Potential Complementary Therapeutic Strategy

**DOI:** 10.3390/nu15173797

**Published:** 2023-08-30

**Authors:** Katrin Witte, Kerstin Wolk, Ellen Witte-Händel, Torben Krause, Georgios Kokolakis, Robert Sabat

**Affiliations:** 1 Psoriasis Research and Treatment Center, Charité—Universitätsmedizin Berlin, Corporate Member of Freie Universität Berlin and Humboldt-Universität zu Berlin, 10117 Berlin, Germany; 2 Interdisciplinary Group of Molecular Immunopathology, Dermatology/Medical Immunology, Charité—Universitätsmedizin Berlin, Corporate Member of Freie Universität Berlin and Humboldt-Universität zu Berlin, 10117 Berlin, Germany; 3 Inflammation and Regeneration of Skin, BIH Center for Regenerative Therapies, Charité—Universitätsmedizin Berlin, Corporate Member of Freie Universität Berlin and Humboldt-Universität zu Berlin, 13353 Berlin, Germany

**Keywords:** acne inversa, metabolic syndrome, obesity, hypertension, dyslipidemia, NAFLD, hyperglycemia, polyphenol, *Olea europea*, *Withania somnifera*, *Vitis vinifera*, *Camellia sinensis*

## Abstract

Hidradenitis suppurativa (HS) is a chronic inflammatory disease characterized by the appearance of painful inflamed nodules, abscesses, and pus-draining sinus tracts in the intertriginous skin of the groins, buttocks, and perianal and axillary regions. Despite its high prevalence of ~0.4–1%, therapeutic options for HS are still limited. Over the past 10 years, it has become clear that HS is a systemic disease, associated with various comorbidities, including metabolic syndrome (MetS) and its sequelae. Accordingly, the life expectancy of HS patients is significantly reduced. MetS, in particular, obesity, can support sustained inflammation and thereby exacerbate skin manifestations and the chronification of HS. However, MetS actually lacks necessary attention in HS therapy, underlining the high medical need for novel therapeutic options. This review directs attention towards the relevance of MetS in HS and evaluates the potential of phytomedical drug candidates to alleviate its components. It starts by describing key facts about HS, the specifics of metabolic alterations in HS patients, and mechanisms by which obesity may exacerbate HS skin alterations. Then, the results from the preclinical studies with phytochemicals on MetS parameters are evaluated and the outcomes of respective randomized controlled clinical trials in healthy people and patients without HS are presented.

## 1. Hidradenitis Suppurativa

**Figure 1:**
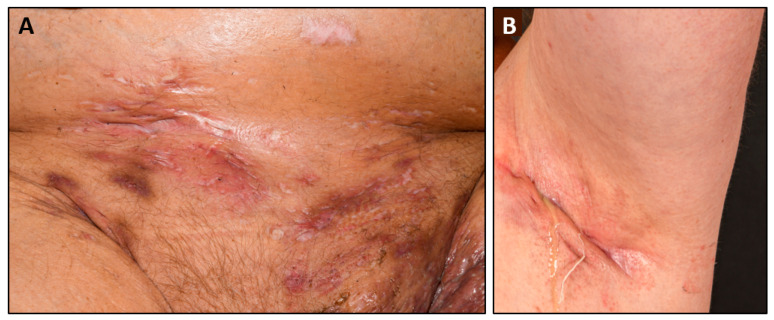
Representative picture of axillary (**A**) and lower belly/inguinal (**B**) skin lesions of HS patients.

Hidradenitis suppurativa (HS) is a chronic inflammatory disease affecting the intertriginous skin, particularly at the axillary, inguinal, gluteal, and perianal sites [1]. Painful inflamed nodules, abscesses, and pus-draining sinus tracts recur in these areas of the skin. In addition, destructive skin remodeling processes in the course of the disease lead to scars that restrict movement (Figure 1). This debilitating disease usually starts in early adulthood and shows an estimated worldwide prevalence of about 0.4–1% [1,2,3,4,5,6]. 

After the onset of the first symptoms, the diagnosis of HS still takes about 10 years on average, a fact that is important because the disease duration correlates with the number of comorbidities of respective patients [7]. Despite the regional differences, men appear to be as equally affected as women when viewed globally [8,9,10,11,12,13]. Given the great physical and mental burden of the disease, it is not surprising that HS patients have been found to show a considerable reduction in quality of life parameters, and this reduction is even more pronounced compared to other chronic inflammatory skin diseases, including psoriasis or atopic dermatitis [14]. Anxiety, depression, body image impairment, and passive forms of indirect self-destructiveness together with stigmatization and social exclusion or self-isolation are additional aspects frequently associated with HS [15,16,17,18,19,20]. Furthermore, owing to a reduced employment rate and an increased absenteeism and presenteeism, HS leads to a significant loss of national gross value added and, therefore, is of great socio-economic importance [21].

Known predisposing factors for HS include genetic as well as lifestyle factors [22]. Among the lifestyle factors, obesity and smoking, frequently met in HS patients, were linked to the development of skin alterations. However, the mechanism of lesion development is still not fully understood, especially the initial events triggering the disease, which are still unclear. It is assumed that obesity supports a subclinical inflammatory milieu around the hair follicle in apocrine gland-bearing intertriginous skin [22]. In the early stage of HS, epidermal hyperplasia, including acanthosis and hyperkeratosis, leads to infundibular alterations promoting follicular occlusion, whereby secreted inflammatory mediators (e.g., cytokines) from infiltrated mononuclear immune cells may account for this process. Nicotine might contribute to these alterations by promoting epidermal hyperplasia and altering the skin microbiome [23,24,25]. Resulting retention of sebum within the hair follicle then leads to its dilatation, propagation of bacteria, and inflammation [26]. Thus, bacterial components and alarmins released from damaged follicular cells are sensed by local immune cells through pattern recognition receptors, provoking high immune cell infiltration and the formation of inflamed nodules and abscesses [22,26,27]. The continuous cross-talk of cutaneous tissue cells with those activated immune cells, in particular, macrophages, T cells, B/plasma cells, and neutrophilic granulocytes, results in the secretion of further pro-inflammatory cytokines and matrix-degrading enzymes (matrix metalloproteinases), which drive skin destruction and allow for the formation of pus-draining sinus tracts in the chronic stage of HS [27,28,29,30,31,32,33,34,35,36,37]. HS lesions also contain high levels of the anti-inflammatory cytokine interleukin (IL)-10 [26,38]. Interestingly, the long-term effects of bacterial products on monocytes and macrophages are very similar to the effects of IL-10 on these cells [39]. Finally, extensive scarring can develop as the result of the ongoing tissue-remodeling processes. Immune mediators produced locally in HS lesions can enter the circulatory system and act in other organs, promoting the occurrence of comorbidities [27,32,34].

## 2. Metabolic Alterations in HS

An important clinical aspect associated with HS is the presence of profound metabolic alterations of those affected, including central obesity, hypertriglyceridemia, hypo-high-density lipoprotein (HDL) cholesterolemia, hyperglycemia, and hypertension [40]. When three of these criteria are met, the diagnosis of metabolic syndrome (MetS) can be confirmed. The number of fulfilled MetS criteria typically increases with age, whereas this observation does not apply to HS patients. In fact, already in early life (≤34 years of age), 40% of HS patients are shown to be affected by the MetS compared to 0% in age-matched controls [40]. In addition to MetS, HS is associated with numerous additional comorbidities, including spondyloarthritis, inflammatory bowel disease, non-alcoholic fatty liver disease (NAFLD), and cardiovascular disease [41,42,43,44,45,46,47,48]. 

Among MetS criteria, central obesity is the most frequent found in ~65% of HS patients (compared to 24% in the healthy controls) and is assumed to play a pathogenetic role in HS [40]. Adipose tissue is able to adapt to times of varying nutrient availability through releasing free fatty acids (FFAs) from stored triglycerides during times of nutrient shortage and through the storing of triglycerides in times of caloric excess [49]. If the physiological storage is at its limit, adipose hyperplasia (increase in cell numbers) occurs. This is associated with decreased blood perfusion, local immune cell activation, apoptosis, and enhanced mechanical stress due to the tightness of cells within the adipose tissue. In contrast to the so-called metabolically healthy obesity (MHO), where the majority of adipose tissue is located in subcutaneous depots, metabolically unhealthy obesity (MUHO) leads to central obesity (visceral adiposity), with triglycerides predominantly deposited in ectopic sites, including visceral adipose tissue or inner organs (e.g., the liver, skeletal muscle, and heart) [49]. 

## 3. Proinflammatory Mechanisms of Obesity

Obesity might affect HS in four different ways: at the physical, microbial, immunological, and metabolic levels [22]. First, obesity leads to enlarged skin folds that may support lesion development through continued wetness, maceration, increased mechanical friction, and injury. Second, resulting anaerobic conditions in those skin folds in turn provide the basis for the altered microbiome pattern observed in HS patients. Third, hypertrophic adipose tissue mediates low-grade systemic inflammation through pro-inflammatory mediators (e.g., cytokines and chemokines) secreted by immune cells within the adipose tissue and induces oxidative stress, both of which worsen the skin condition as well as HS comorbidities [50,51]. In comparison to normal-weight individuals, hypertrophic adipose tissue contains increased numbers of neutrophils and macrophages, known to play an important role in the development of low-grade systemic inflammation [52,53]. From the in vivo models, it can be deduced that neutrophils, infiltrated into the hypertrophic adipose tissue in the early stage of obesity, mediate the recruitment and polarization of macrophages through the increased secretion of proinflammatory as well as macrophage recruiting chemotactic mediators (e.g., IL-6, tumor necrosis factor (TNF)-α, CCL2) [54,55]. Furthermore, the tight cross-talk of neutrophils and adipocytes was suggested to mediate NLRP3 inflammasome activation, the inflammation of adipose tissue, and biasing of neutrophils towards a hyperinflammatory state [56,57]. Fourth, adipose tissue is not only an energy reservoir, but also a major endocrine tissue [49,58]. In HS, the pattern of adipokines, peptide hormones derived from adipose tissue that regulate metabolic processes, e.g., insulin sensitivity (regulated by adiponectin) and body weight homeostasis (regulated by leptin) are dysregulated [59,60,61]. In fact, the serum level of anti-inflammatory adiponectin clearly decreases, whereas leptin levels increase in HS compared to healthy donors, indicating the presence of a leptin resistance, which further promotes obesity [59,60]. As immune cells are also directly targeted by adipokines, the altered adipokine pattern might contribute to the development of a pathogenetic immune–metabolic circuit in HS patients [58,62]. Interestingly, the metabolism of blood CD4^+^ T cells appears to also be altered in HS patients. In fact, it was recently found that the expression of several genes involved in oxidative phosphorylation was downregulated in the blood CD4^+^ T cells of HS patients and a few transcripts for glycolysis-dependent energy production were increased [63]. 

It should be noted that obesity is associated with the enhanced systemic level of FFAs [49]. FFAs negatively impact glucose and lipid metabolism, being risk factors for developing insulin resistance and dyslipidemia [49]. Dyslipidemia and hyperglycemia, in turn, are risk factors for developing cardiovascular diseases [49,64,65,66]. Of note, FFAs may also promote inflammation by binding to TLR4 on monocytic immune cells and induce NLRP3-dependent IL-1β production [67,68,69]. As FFAs are released from visceral depots directly into the portal circulation, FFAs also affect liver homeostasis and promote the development of NAFLD [49]. Accordingly, higher prevalences of hyperglycemia, dyslipidemia, cardiovascular alterations, and NAFLD in HS patients compared to the controls was reported [40,41,43,44,46]. In fact, ~26% of HS patients were found to suffer from hyperglycemia compared to 8% in the healthy controls, and the incidence of diabetes increased at least two-fold in HS patients [40,70]. Aspects of dyslipidemia are found in 50% (hypo-HDL cholesterolemia) and 38.8% (hypertriglyceridemia) of HS patients, compared to 18% and 22%, respectively, in the healthy controls [40]. Furthermore, ~70% of HS patients compared to 30% of the controls were also affected by NAFLD [43,46]. The described mitigating impact of bariatric surgery and weight loss on HS severity supports the concept of the significant contribution of adipose tissue to the cutaneous inflammation in HS patients [71,72]. Furthermore, the relevance of inflammation to MetS is supported by the observation that anti-inflammatory therapy targeting TNF-α may improve MetS severity in patients with rheumatoid arthritis [73]. However, the respective data for HS patients are lacking at present. Overall, the strongly increased presence of metabolic alterations and its sequelae in HS patients are serious risk factors contributing to the substantially reduced life expectancy [74]. In fact, HS patients lived an average of 14.7 years less than the controls, with cardiovascular disease being their leading cause of death [74]. 

## 4. HS Therapy: Time for a New Perspective?

Therapeutic options for moderate to severe HS include, at present, long-term antibiotic treatment and the surgical excision of skin lesions, whereby these therapies do not result in a sustained improvement of the disease-associated reduced quality of life of patients [14,75]. Furthermore, at present, there are only two approved immune therapies for HS:, the TNF-α-neutralizing antibody adalimumab and the IL-17A-neutralizing antibody secukinumab. In contrast to psoriasis, only a proportion of HS patients benefits from these biologicals. The limited treatment options and consideration that a relevant portion of HS patients refuse single-therapy elements or have relevant contraindications demonstrate the great need for novel and innovative therapeutics for HS treatment [1,76]. The inclusion of MetS comorbidities in therapy concepts, in particular, obesity, as a relevant trigger factor for HS symptoms is also still insufficient. Based on the increased prevalence of mood disorders among HS patients, lifestyle changes are also difficult to realize for those patients. To close this gap, phytotherapy appears to be an appropriate complementary therapeutic approach by targeting MetS elements. In fact, there is a positive perception of alternative therapeutics among both HS patients and dermatologists [77,78]. In this review, we evaluate the potential impact of selected phytomedical drugs on MetS parameters. In this way, we hope to identify candidates that can be tested in future studies on HS patients, applied in daily practice, and complement HS therapy in the long term. 

## 5. Phytotherapy—A Therapeutic Concept with a Long History

The traditional use of herbal plant-based medicine has a very long tradition that dates back several thousand years [79]. The first descriptions of the use of herbal medicine was found on Sumerian clay slabs from Mesopotamia (~3000 BC) and Egyptian papyrus rolls (~1550 BC) [79]. Medicinal plants are also mentioned in the Bible, another old scripture. In the traditional medicine of ancient Greece, the use of locally growing herbs for medical purposes also played an important role and was proposed by Hippocrates (460–377 BC) [79]. The Shen Nong Ben Cao Jing is another early written record (date of origin unclear: 25–220 AD) describing a variety of medical plants and their therapeutic uses according to traditional Chinese medicine [80]. Later, the monastic medicine (i.a. represented by Hildegard von Bingen, 1098–1179) led to the widespread distribution of herb knowledge among the local population. In the 16th century, Paracelsus (1493–1541) laid the foundation for the concept of spagyric medicine, a term derived from the Greek words “spien” (separate) and “agera” (unite) [81]. Two-hundred years later, Carl von Linne finally developed a binary nomenclature for plants that brought the needed system into the plant kingdom [82]. The term phytotherapy was coined by the French physician Henri Leclerc (1870–1955) and comprises the topical application or internal medical administration of plants or herbs. These include their use in the native or processed form as decoctions, extract preparations, or isolated key substances. To date, the European Medicines Agency (EMA) database of the Committee on Herbal Medicinal Products (HMPC) already lists 167 completed monographs for phytomedical plants (https://www.ema.europa.eu/en/medicines, accessed on 7 August 2023).

## 6. Potential Phytotherapeutic Options for MetS in HS Patients

**Figure 2:**
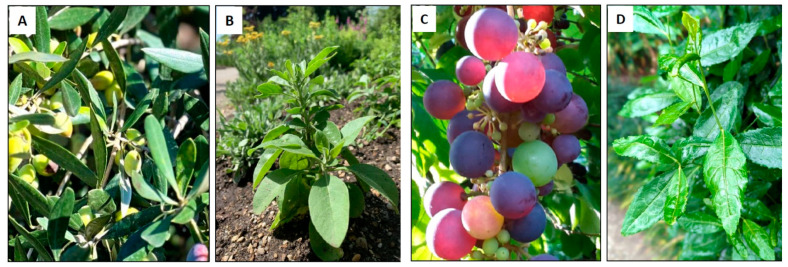
Botanical pictures of *Olea europea* (**A**), *Withania somnifera* (**B**), *Vitis vinifera* (**C**), and *Camellia sinensis* (**D**).

Targeting metabolic alterations by phytochemicals might be a complementary therapeutic strategy of HS. Especially, their anti-inflammatory, antioxidative, glucose, lipid metabolism regulating, and their described cardio- and hepatoprotective properties are potentially interesting in this regard [83,84]. For this review, phytochemicals were selected within the field of phytotherapy according to the availability of the preclinical and clinical data on a single metabolic syndrome parameter. Accordingly, *Olea europea*, *Withania somnifera*, *Vitis vinifera*, and *Camellia sinensis* were found to represent appropriate candidates for the indication of MetS (Figure 2). As the effects of phytochemicals on primary and tumor cells are different [85,86], the preclinical data based on primary cells were primarily evaluated in this review. Furthermore, regarding the available human in vivo data, only placebo-controlled, blinded, randomized clinical trials (RCTs) were presented in this review.

### 6.1. Olea europea

As an important agricultural plant, different parts of the olive tree are used for nutritional and medical purposes, the leaves, olive fruits, and olive oil also being elements of the so-called Mediterranean diet. The main active constituents of olive oil fruits as well as of olive leaf extract (OLE) are the polyphenols oleuropein (secoiridoid) and hydroxytyrosol (phenylethanoid), which is also generated through the metabolization of oleuropein. Several preclinical in vitro and in vivo studies investigating the mode of action of these substances implied the beneficial effect on metabolic dysfunction. In fact, oleuropein and hydroxytyrosol were shown to prevent LDL oxidation and strengthen endogenous antioxidative and arteroprotective mechanisms, reducing endoplasmic reticulum stress and platelet aggregation in vitro [87,88,89,90,91,92,93,94]. 

In vivo, *Olea europea* leaf-derived phytochemicals improved dyslipidemia, adipokine profile, glucose homeostasis, and antioxidative capacity in several diabetes, oxidative stress, and obesity animal models [91,95,96,97,98,99,100,101,102,103,104]. In line with these studies, these phytochemicals ameliorated high-fat-diet-induced body weight increase and white adipose tissue hypertrophy in vivo in rodent models [96,100,102,103,104]. The reported enhancing effect of hydroxytyrosol and oleuropein on adipocyte lipolysis in vitro might therefore contribute to their normalizing effect on lipid metabolism in vivo [105,106,107]. The data from Vezza et al. and Wang et al. indicate the normalization of obesity-related dysbiosis and the downregulation of inflammatory cytokines as another mechanism underlying the positive impact of the MetS parameter on high-fed diet-induced murine obesity [100,101]. Moreover, an increase in the systemic adiponectin level and upregulation of MAPK, as well as the suppression of PPARg expression in adipose tissue were suggested by Hadrich et al. and Scoditti et al. to underly the anti-obesity effects of olive leaf phytochemicals [96,108]. 

Furthermore, an improvement of cardiovascular parameters by hydroxytyrosol using an in vivo diabetes rat model was demonstrated [109]. A cardioprotective role was also suggested for olive leaf phytochemicals using in vivo animal models of high-fat-diet-induced metabolic syndrome, diabetes, arteriosclerosis, and ischemia [104,110,111,112,113,114,115,116]. The underlying mechanism of cardioprotection might involve nitric oxide-mediated vasodilatation and oxidative stress reduction [104,115,117]. Moreover, according to the in vitro data, olive leaf phytochemicals were observed to show anticoagulative properties in healthy and experimentally induced ischemic rodents [87,89,111,118]. 

For the described hepatoprotective effect of oleuropein and hydroxytyrosol in high-fat-diet-based in vivo rodent models, an attribution to the normalization of hepatic PPARg, Nrf2, and NF-kB pathway activity was suggested [101,102,104,119,120]. 

The available RCTs on the evaluation of olive leaf extract (OLE), oleuropein, or hydroxytyrosol indicated an attenuating effect on the parameters of MetS, confirming in part the preclinical study data (Table 1). As the individual contribution of containing fatty acids and polyphenols of olive oil to the observed effects in respective RCTs as challenging, only RCTs using OLE, oleuropein, or hydroxytyrosol were considered for the evaluation and were discussed here. RTCs evaluating the potential of OLE on glucose metabolism did not present a consistent picture yet hinted at some beneficial effects contributing to the normalization of glucose homeostasis. In fact, OLEs were found to reduce the postprandial plasma glucose level of healthy, obese, pre-hypertensive, or osteoporosis participants after single or long-term applications in 3 of 4 RCTs evaluating this outcome measure [121,122,123,124]. However, fasting glucose levels were not affected by long-term OLE applications [125,126,127]. A short-term treatment of healthy participants with oleuropein followed by glucose tolerance testing in the absence of oleuropein also did not influence the post-prandial blood glucose level [128]. In contrast, insulin sensitivity and pancreatic β-cell function were improved in obese participants by OLE [122]. However, in obese or hypertensive participants, insulin levels postprandially decreased [122] or remained unchanged [125,127] after long-term OLE treatment; an increase was assessed after a single application in healthy study cohorts [121,124]. Based on the increase of the hormone GLP-1 that supports insulin secretion with a concurrent reduction in its inhibitor DPP-4, an antidiabetic property was suggested for OLE by Carnevale et al. [121]. In contrast to the preclinical studies, the data from respective RCTs regarding the effects of OLE on the lipid profile were not consistent. In two out of four studies evaluating lipid parameters, an improvement of dyslipidemia parameters, including a reduction in total cholesterol (CH), low-density lipoprotein (LDL), and triglyceride (TG) levels after long-term applications, were reported [126,129]. Whether *Olea europea* phytochemicals might be beneficial in body weight management is not yet clear. The data on the antioxidative capacity of *Olea europea* phytochemicals from RCTs are sparse. Only one RCT investigated this parameter and found a decrease in postprandial oxidative stress in healthy participants after a single oleuropein application [121]. Regarding the cardiovascular measures, no clear influence of OLE on the blood pressure parameter was found, whereas a slight reduction in systolic and diastolic blood pressure levels was reported by Lockyer et al. after a 6-week OLE application in pre-hypertensive participants; no influences on blood pressure was described by Stevens et al. and de Bock et al. after 8- or 12-week OLE treatments, respectively [122,126,127]. A head-to-head study investigating the effect of OLE and captopril on blood pressure in hypertensive participants revealed a comparable effectivity for both substances [130]. However, the lack of a placebo group and the use of only low-dose captopril were certainly limitations of the study. The impact of OLE treatment on vascular function was also not clear as the short- and long-term studies showed inconsistent data [126,131].

### 6.2. Withania somnifera

The winter cherry is a common plant predominantly found in Mediterranean regions, with a long history of use in ayurvedic medicine. Among withanolides, secondary phytochemicals present in the root of withania somnifera, withaferin A (steroidal lactone), are the most studied component. Data obtained from the preclinical studies evaluating the effects of withaferin A in vitro and in vivo suggest the antidiabetic, anti-obesity, anti-oxidative, and anti-inflammatory potential of this substance.

In vitro, withaferin A caused an improvement of glucose metabolism, enhanced insulin secretion by pancreatic β-cells, and mediated the protection of pancreatic islet cells against inflammatory cytokine-induced cell death [132,133]. Moreover, the inhibition of adipogenesis by withaferin A was also observed in vitro [134]. Furthermore, in a palmitic acid-induced oxidative stress in vitro model, withaferin A inhibited ROS and inflammatory cytokine production, whereas it restored the impaired insulin signaling and NO production in endothelial cells [135].

In line with the in vitro data, withaferin A was also described to show antidiabetic activity in vivo. In fact, an improvement of insulin resistance, glucose metabolism, and adiponectin level was observed using respective in vivo murine obesity and diabetes models [134,136,137,138]. The suggested underlying mechanisms included the regulation of genes involved in the insulin and PPARγ pathway [134]. 

Furthermore, withaferin A ameliorated body and adipose tissue weight gain and improved the lipid profile in various murine obesity models [134,136,137,139,140,141]. In line with these observations, withaferin A was identified to act as a leptin sensitizer and inhibit the food restriction-based reduction in basic energy expenditure in obese mice [141]. Furthermore, withaferin A-induced browning of white adipose tissue accompanied by enhanced mitochondrial activity observed in high-fat-diet-fed mice might contribute to its anti-obesity effects [139,140,142]. Accordingly, sympathetic denervation reduced withaferin A-mediated white adipose tissue browning and a decrease in obesity indicated the important role of the sympathetic nerve/adipose axis involving PRDM16 and FATP1 [139]. 

Furthermore, the data from in vivo rat models of hypertension, ischemia reperfusion injury, and cardiac toxicity suggest the cardioprotective properties of *Withania somnifera* phytochemicals [143,144,145,146,147]. An enhanced oxidative stress reduction was suggested as one mechanism underlying these findings. The amelioration of hepatic steatosis and normalization of liver enzymes, hepatic inflammatory markers (IL-6, TNF-α, IL-1β, CRP, MCP-1, COX2), endogenous antioxidant system molecules, and regulated enzymes involved in lipid and glucose metabolism in vivo using a high-fat-diet-induced murine obesity model also implied the hepatoprotective potential of withaferin A [134,136,137]. The withaferin A-dependent improvement of steatohepatitis in leptin-signaling-deficient ob/ob mice thereby suggested leptin-independent mechanisms for hepatoprotection [148]. In a further study of murine diet-induced obesity, the hepatoprotective effect of withaferin A was suggested to be related to the direct activation of liver X receptor a/farnesoid X receptor (LXRα/FXR) [137]. Additionally, the data from a murine liver toxicity model reveals that withaferin A is able to reduce liver injury in vivo [149]. This effect was suggested to be attributed to the induction of Nrf2 and genes of the antioxidative glutathion system [149]. The therapeutic hepatoprotective potential of withaferin A was also shown using an in vivo hepatitis model that revealed the effective attenuation of D-galactosamine/LPS-induced liver damage by this phytochemical [150]. The authors suggested the limitation of macrophage NLRP3 activation and IL-1β secretion as possible mechanisms of action of withaferin A in this model [150]. 

To date, there is limited data on the three available placebo-controlled RCTs on the influence of withania somnifera root extract (WSRE) on the features of metabolic syndrome (Table 2). In chronic, stressed, overweight participants, the application of WSRE provoked a reduction in food craving and perceived stress scores as well as serum cortisol level, whereas the assessed happiness score increased [151]. In line with these data, the body fat percentage of healthy participants undergoing resistance training was more efficiently reduced under WSRE treatment [152]. In healthy athletes, WSRE treatment improved the cardiorespiratory endurance and increased the antioxidative capacity [153]. Moreover, WSRE showed effectivity in improving hypothyreosis. In fact, WSRE treatment led to a significant reduction in TSH and a concomitant increase in triiodothyronine (T3) and thyroxine (T4) levels [154]. The results from three further completed RCTs evaluating the effect of withania somnifera on weight loss and steatohepatitis are expected in the near future (clinicaltrials.gov). Furthermore, as WSRE was tested more extensively for other indications, there were substantial data on pharmacokinetics and safety [151,154,155,156,157,158,159]. 

### 6.3. Vitis vinifera

The beneficial properties of wine grapes on human health are not only appreciated in Mediterranean regions, and this is displayed by the extensive research on this topic. Among the range of phytochemicals, resveratrol (phytoalexin) is found in the peel and pulp, whereas the seeds mainly contain polyphenols (proanthocyanidins and flavonoids). Especially for grape seed polyphenols, the potential impact on the metabolic features was described. 

In fact, in addition to the anti-inflammatory and antioxidative effects, grape seed extract (GSE) was found to regulate genes involved in metabolic homeostasis in vitro [160,161,162]. Moreover, GSE was reported to inhibit adipogenesis and increases lipolysis via targeting PPARγ in vitro [163,164]. Additionally, using endothelia cells as well as aortic ring cultures, GSE treatment revealed the eNOS-dependent vasodilatative potential in vitro [165,166].

Accordingly, the data from preclinical in vivo studies confirm the glucose and lipid metabolism-regulating as well as hepato- and cardioprotective potential of GSE. Indeed, GSE treatment improved the insulin resistance in in vivo models of obese and fructose-rich-diet rodents and attenuated pancreatic degeneration in a diabetes model [167,168,169,170,171,172,173]. In another study using healthy rats, GSE treatment following glucose intake was found to modulate glucose metabolism by upregulating the incretin GLP-1 and downregulate the GLP-1 inactivating enzyme DPP-4 [160,161,174]. Furthermore, GSE might protect pancreatic b-cell function from lipotoxic stress in vitro and in vivo in Western-diet-fed rats [175]. 

Moreover, weight gain, fatty liver, adipokine level, and lipid profile were counteracted in vivo in obese or fructose-fed rodents by GSE treatment [167,168,169,176,177,178,179,180,181]. The influence of GSE on weight gain might be related to an increase in portal GLP-1, ghrelin, and decreased cholecystokinin levels, reducing gastric emptying combined with enhanced satiety and reduced food intake [182]. The alleviating effect of GSE on cholesterol levels might be associated with increased bile acid secretion and the upregulation of the cholesterol-metabolizing enzyme CYP7A1 [183]. Metabolic improvements by GSE in obese mice might in part also be related to the upregulation of thermogenesis and adipose tissue browning marker UCP1, BAT and PRDM16 in white adipose tissue, and the improvement of intestinal GLP-1 and DPP-4 expressions [160,161,174,184]. The attenuation of the obesity-induced upregulation of miR-96 and its target mTOR might also contribute to GSE-mediated metabolic improvements in obesity [178]. Furthermore, Pascula-Serrano et al. suggested the GSE-mediated expansion of healthier visceral adipose tissues in obese rats as a mode of action in this model [177]. The normalization of dysbiosis might be a further mechanism of GSE-mediated metabolic improvements and attenuated obesity [173,181,184].

In addition to its protective role in obesity and dyslipidemia, GSE was also assumed to have cardioprotective properties. Indeed, GSE provoked the obesity-related prevention of cardiac siderosis, improvement of ischemia-related cardiac dysfunction and remodeling, attenuation of hypertension-dependent arterial remodeling, as well as protection against toxicity-induced cardiac damage in respective rodent in vivo models [176,185,186,187]. Furthermore, the hepatoprotective role of GSE was suggested based on the results from an in vivo rat NAFLD model, whereby GSE was found to be more effective than metformin [180]. The PPARγ-dependent modulation of hepatic lipid metabolism might be one mechanism underlying the protective effects of GSE on metabolic parameters [188].

To date, a range of RCTs evaluating the clinical potential of GSE on metabolic syndrome features were performed (Table 3). Three RCTs evaluated the impact of GSE on glucose metabolism and overall showed a limited effectivity [189,190,191]. However, in one of these studies, a GSE-mediated improvement of insulin sensitivity (HOMA-IR) was reported; there was no impact on fasting glucose levels but a decreased fructosamine level was reported by another study [189,190]. Furthermore, after long-term GSE treatment, only a tendency for improved fasting glucose and insulin sensitivity (HOMA-IR) was observed by Park et al. [191]. According to the preclinical data, GSE was shown to have a positive impact on the lipid profile parameters of dyslipidemia and overweight participants and heavy smokers [192,193,194,195]. In fact, long-term treatment resulted in reduced total cholesterol [192,193,194,195], LDL [192,193,194] and triglyceride levels [192,195]. Moreover, a GSE-dependent reduction in the artherogenic index of plasma (AIP) was reported by Yousefi et al. [195]. In contrast, no influence on the lipid parameters was observed in two additional studies [191,196]. Results from 4 RCTs suggest the therapeutic use of GSE for body weight management. In one study, GSE treatment for 3 days reduced the 24 h energy intake in the subgroup with an increased basal energy requirement of ≥7.5 MJ/day among the healthy participant cohort [197]. Furthermore, greater reductions in body weight and BMI, waist circumference, and waist to hip ratio of obese participants undergoing a caloric-restriction diet were observed when concomitantly treated with GSE in the long term [198]. Yousefi et al. also found a reduced visceral adiposity index (VAI) in GSE compared to placebo-treated overweight participants on a calorie-restriction diet [195]. Moreover, in postmenopausal women, long-term GSE treatment resulted in significantly heavier muscle mass [199]. An improved endogenous antioxidative capacity [189,194], reduced inflammatory markers (TNF-α, CRP) [198], as well as perceived stress [200], anxiety, and depression scores [199] were described in single RCTs evaluating GSE effects on T2D, healthy smokers, obese, hypertensive, and postmenopausal participants, respectively. Regarding the cardiac parameters, the available data obtained from respective RCTs reveal that the long-term GSE treatment of prehypertensive, mild hypertensive, and postmenopausal participants results in decreased systolic and, in some studies, diastolic blood pressure levels [191,199,200,201]. In two further RCTs, improvements in blood pressure were even measured after a single GSE application in overweight and prehypertensive participants [202,203]. However, no influence of GSE treatment on blood pressure in hypercholesteremia and pre/stage-I hypertensive participants was reported by Ras et al. or Preuss et al. [196,204]. Considering the vascular parameters, no relevant influence on the vasoactive systemic marker level, endothelial function, and flow-mediated dilatation (FMD) was observed after the long-term treatment of pre/stage-I hypertensive and type 2 diabetic participants with GSE [189,191,201,204]. In contrast, an improvement of the vascular health index of heavy smokers after long-term GSE application was described by Weseler et al. and suggested to be associated with the induced increase in endogenous antioxidative potential [194]. Furthermore, the overall cardiac output, assessed by impedance cardiography, was improved after the application of a single GSE dose in obese, but not in healthy, participants [202]. 

### 6.4. Camellia sinensis 

The tea plant (*Camellia sinensis*) is found in tropical and subtropical areas with a long history in agricultural use for tea preparation that spans over 1500 years. The main polyphenolic constituents of *Camellia sinensis* are catechins (flavan-3ols) and their derivatives. Among these, epigallocatechine-3-gallate (EGCG) as well as a whole polyphenol mixture prepared as green tea extract (GTE) are the most studied phytochemicals of *Camellia sinensis*. 

Furthermore, potential antidiabetic properties, including the improvement of blood glucose level and insulin resistance, were suggested for tea catechins on the basis of several preclinical in vivo studies [205,206,207,208,209,210,211]. Accordingly, EGCG was found to provoke a decrease in intestinal glucose absorption [205,212]. Regarding its mode of action, it was suggested that the inhibition of α-amylase α-glucosidase activity as well as the activation of NRF2 signaling and the regulation of glucose transporters might contribute to the antidiabetic effects of EGCG [205,209,213,214]. Whether EGCG influences the tissue uptake of blood glucose is not clear, to date, as there are opposing data on this idea [205,212]. Furthermore, tea catechins might increase blood glucose levels when pre-prandially administered and when already systemically present at the time of glucose-tolerance testing [212].

In high-fat-diet-induced rodent obesity models, EGCG targeted a further metabolic syndrome feature, as it ameliorated dyslipidemia in vivo [208,211,215,216,217]. Furthermore, a decrease in body weight and body fat mass in response to EGCG treatment was observed in vivo [207,208,211,215,218,219,220]. The inhibition of transcriptional activators regulating the expression of proprotein convertase subtilisin/kexin type 9 (PCSK9), thereby increasing hepatic LDL uptake, was discussed as a possible mechanism underlying the LDL lowering effects of EGCG [221]. The upregulation of adipocyte autophagy and regulation of thermogenic and adipogenic genes is a hypothesized mechanism underlying the weight-reduction properties of EGCG [218,219,222,223]. 

In addition to its anti-obesity and anti-diabetic potential, cardioprotective properties have been postulated for EGCG as well. Using hypoxia-reperfusion injury, diabetes, atherosclerosis, and endothelial dysfunction in vivo rodent models, EGCG was found to ameliorate cardiovascular parameters and endothelial dysfunction [206,224,225,226]. Interestingly, the suppression of eNOS uncoupling, a process that is associated with oxidative stress-induced endothelial dysfunction, by the normalization of BH4 level was identified as a possible underlying mechanism [227,228]. Additionally, the functional inhibition of OMA-1, a metalloendopeptidase that negatively affects mitochondrial function, as well as the inhibition of the mitochondrial apoptosis pathway by EGCG, was suggested to improve cardiomyocyte function [206,229]. 

Using a bile duct ligation-based liver injury, combined obesity and hypertension, as well as NAFLD in vivo models, the hepatoprotective role of tea catechins was further proposed [217,230,231,232]. 

To gain further insights into and to evaluate the clinical potential of polyphenols from *Camellia sinensis*, a variety of RCTs focusing on metabolic syndrome parameters were performed (Table 4). The evaluation of the data obtained from respective RCTs reveal that, in terms of glucose metabolism, tea polyphenols might play a bivalent role. Where some studies showed an improvement in glucose level, insulin sensitivity, and HOMA-IR index in healthy or obese participants [233,234,235,236,237,238], others did not find a positive influence of tea catechins on glucose metabolism [239,240,241,242,243]. In a further RCT, a decrease in fasting insulin after the long-term decaffeinated GTE treatment of obese participants was detected only in the subgroup showing baseline insulin levels ≥ 10 µIU/mL [244]. Of note, the timing of the application might account for the catechin-dependent outcome on the glucose level. In fact, in an open randomized clinical trial, the treatment of healthy participants with green tea catechins one hour before glucose-tolerance testing resulted in higher plasma glucose levels, whereas a reduction in glucose levels was observed when catechins and glucose were concomitantly administered [212]. However, based on the limitation of the available data on the latter issue, a final assessment could not be performed at this point. Regarding the influence of tea catechins on the lipid profile, the available data also do not provide consistent results. In 5 out of 10 evaluated placebo-controlled double-blind RCTs, an improvement of single, but not all, assessed lipid profile parameters, including a decrease in total cholesterol, LDLs, and triglycerides by GTE and EGCG in healthy, obese, and diabetic participants was reported [234,245,246,247,248]. In contrast, no impact of long-term EGCG or GTE treatments on these parameters in obese or postmenopausal participants was found [239,241,242,243,249]. Body weight reduction was observed in only one RTC after the long-term treatment of metabolic syndrome participants [250]. In a further study, a GTE-dependent increased fat oxidation in a healthy study cohort undergoing exercise intervention was observed compared to the exercise intervention group taking a placebo [237]. Moreover, a delayed gastric emptying and increased satiation as well as adiponectin level were found in healthy participants treated with a single dose of EGCG [240]. However, no influence on body weight, BMI, body fat mass, fat oxidation, waist circumference, energy intake, and satiety was reported by GTE and EGCG in obese participants in the majority of the published RCTs [233,235,239,242,249,251,252,253]. Regarding the cardiovascular parameters, reduced arterial stiffness and increased flow mediated dilatation (FMD) after the long-term treatment of coronary artery disease and diabetic participants was reported by Widlansky et al. and Quezada-Fernandez et al. [254,255]. Increased FMD in response to the single application of tea catechins was also observed in a placebo-controlled, but open-label, clinical trial [256]. Furthermore, in obese participants performing physical exercise, a reduction in the resting heart rate was observed [235]. The data from two further RCTs show a reduction in blood pressure parameters in obese participants in response to long-term EGCG treatment [233,239]. In contrast, no clear impact of GTE and EGCG on cardiovascular parameters, including blood pressure and heart rate in hypertensive participants after resistance training, was reported by Arazi et al. [257]. 

## 7. General Aspects of the Future Use of Phytochemicals in HS Patients

Metabolic alterations, in particular, obesity, can support sustained inflammation and thereby exacerbate skin manifestations and the chronification of HS. However, they lack the necessary attention in HS therapy. Considering the data from the evaluated preclinical and clinical studies suggest that phytochemicals from *Olea europea*, *Withania somnifera*, *Camellia sinensis*, and *Vitis vinifera* represent potent candidates for targeting metabolic dysfunction. As the phytochemicals evaluated here have partly overlapping properties, different phytotherapeutic options for the treatment of single metabolic syndrome features central obesity, insulin resistance, triglyceridemia, hypo-high-density lipoprotein (HDL)-cholesterolemia, and hypertension exist. Furthermore, when considering an integrative HS therapy using phytochemicals, the following aspects should be taken into account. First, the priority of MetS parameter(s) that need the relevant improvements should be determined. Second, considering the present concomitant medication of the patient, the relevant potential drug interactions with the phytochemical candidates should be carefully estimated and taken into account for the decision. Third, the decision for the appropriate phytochemical should also depend on the safety profile of the phytochemical of choice, analyzed in regard of the individual clinical condition of the patient. In general, strict medical supervision and monitoring should be prerequisites for performing integrative therapy using phytochemicals. Before and consecutively during therapy with the selected phytochemicals, it is highly recommended to perform a detailed analysis of the relevant physical (e.g., cardiovascular) and laboratory parameters (including indicators of lipid/glucose metabolism, coagulation status, and liver enzymes), as well as HS (e.g., IHS4; [258]) and QoL scoring to enable the careful monitoring of safety, drug interaction, and therapeutic effectivity. 

## 8. Safety and Drug Interaction

Phytochemicals derived from *Olea europea*, *Withania somnifera*, and *Vitis vinifera* showed an overall good tolerability and safety profile during clinical use [122,151,154,155,156,157,159,200,259,260,261,262,263,264,265,266]. Phytochemicals derived from *Camellia sinensis* were extensively studied in regard of their pharmacokinetics and safety, and for the clinical use of EGCG, an upper safe-dosage limit (338 mg for extracts; 704 mg for beverages) was recommended [267]. This recommendation was based on the described liver toxicity as a possible rare adverse reaction resulting from a high bolus-dose application. In contrast, these safety concerns were not raised for the use of beverages produced from the whole leaves or extract of *Camellia sinensis* [267]. However, therapy with *Camellia sinensis* phytochemicals should be avoided for patients with known hepatic dysfunctions. 

The main described potential drug interactions of the phytochemicals evaluated here were those related to cytochrome P450-metabolizing/detoxifying enzymes. In fact, for *Olea europea*-, *Camellia sinensis*-, and *Vitis vinifera*-derived phytochemicals, an interaction with cytochrome P450-detoxifying enzymes was reported [268,269,270,271,272]. As this may influence the pharmacokinetics of concomitantly administered P450-metabolized drugs, the efficacy of concomitant medication and, respectively, associated clinical parameters should be monitored during the treatment with these phytochemicals. Whether WSRE from *Withania somnifera* interacted with cytochrome P450 enzymes was not clarified; however, the precautionary monitoring of the efficacy of concurrent drug medication was also recommended [273,274,275].

Furthermore, phytochemicals from *Camellia sinensis* were found to be inhibitors of the enzyme catechol-o-methyltransferase (COMT), and might therefore modify the detoxification and metabolization of xenobiotics, catecholamines, and catechol estrogens [276]. For patients carrying the low-activity COMT genotype receiving, e.g., levodopa, apomorphine, isoprenaline, catecholamines, micafungin, or estrogen derivates, or those suffering from estrogen dominance, an awareness for potential drug interactions is needed. For patients with known prediabetes/diabetes, the risk-benefit ratio should also be carefully weighted using this medication based on the possible influence on glucose metabolism [205,212]. Whether the epigenetic modifying potential of EGCG has a clinical relevance for patients remains to be investigated. Of note, *Withania somnifera* phytochemicals were observed to improve thyroid function, indicated from a decrease in TSH and increase in triiodothyronine (T3) and thyroxine (T4) levels in subclinical hypothyroid patients [154]. The monitoring of thyroid parameters is therefore recommended for hyperthyroid patients as well as patients receiving L-thyroxin supplementation. For *Olea europea* phytochemicals, the inhibitory property of enzymes that played a role in Alzheimer’s disease progression in vitro was described; however, the clinical relevance of these data remains to be investigated [277]. More detailed information regarding safety and drug interactions are summarized in an previously published review [83]. 

## 9. Recommendations for Integrated Phytotherapy Targeting MetS Parameters in HS Patients

**Figure 3:**
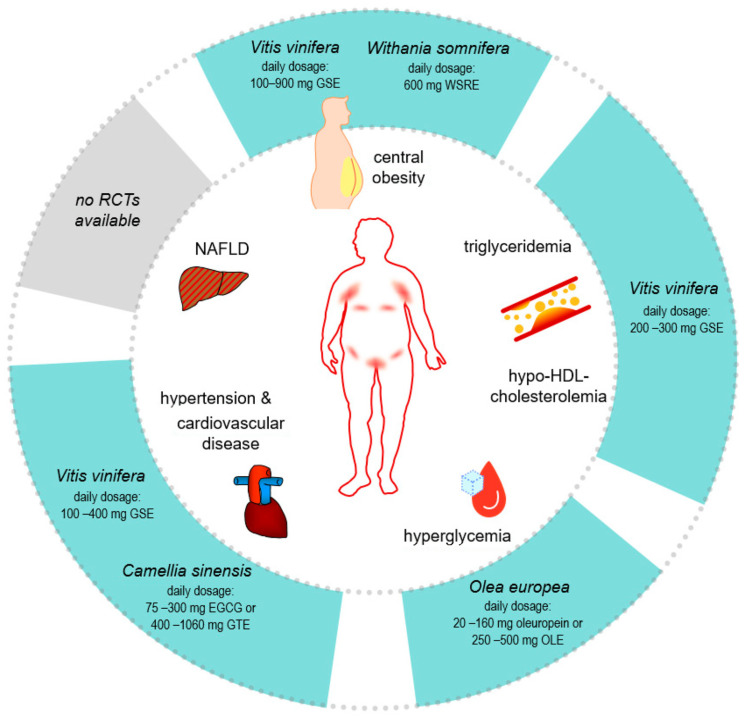
Schematic overview of metabolic alterations frequently observed in HS patients and their potential targeting by *Olea europea*, *Withania somnifera*, *Vitis vinifera*, and *Camellia sinensis*.

For the improvement of glucose metabolism, in principle, OLE (*Olea europea*) was shown to be eligible (Table 5). A daily dose of 20–160 mg of oleuropein or 250–500 mg of OLE is recommended (Figure 3).

In case of dyslipidemia, GSE (*Vitis vinifera*) was reported to be eligible (Table 5), doses ranging from 200–300 mg (GSE) daily were recommended (Figure 3). Protective effects regarding the cardiovascular parameters were described for GSE (*Vitis vinifera*) and EGCG/GTE (*Camellia sinensis*) (Table 5), whereby more RCTs were available for the latter drug. A daily dosage of 100–400 mg (GSE) or 75–300 mg (EGCG) or 400–1060 mg (EGCG/GTE) were recommended (Figure 3).

The evaluated RCTs reveal that, for weight management, WSRE (*Withania somnifera*) and GSE (*Vitis vinifera*) might represent eligible phytochemical drugs (Table 5). There are more data on GSE than for WSRE; however, the data on additional, already completed RCTs evaluating the effect of *Withania somnifera* on weight loss are awaited in the near future (clinicaltrials.gov). A daily dosage of 600 mg (WSRE) or 100–900 mg (GSE) was recommended (Figure 3).

To date, no RCTs are available evaluating the possible hepatoprotective effects of OLE, WSRE, GSE, and EGCG/GTE (Table 5). Nevertheless, for all these drugs, an improvement of hepatic parameters in various in vivo animal models was reported. However, as described in the above section (Safety and Drug Interaction), EGCG/GTE application is not recommended for patients with hepatic dysfunctions as a safety precaution.

## Figures and Tables

**Table 1:** Characteristics and main study outcomes of placebo-controlled, randomized clinical trials investigating the effects of *Olea europea* on metabolic parameters.

Study Medication	Study Type	Dose Regimen	Cohort Size (*n*)	Study Cohort Criteria	Main Study Results Verum vs. Control (Increased: ↑; Decreased: ↓; Unaffected: ≈)	Ref.
500 mg OLE	pc, db, RCT	daily application (8 weeks)	placebo: 38 verum: 39	overweight participants age: 56 ± 10 years BMI: 29 ± 2.7	≈ fasting glucose, insulin ≈ SBP, DBP ≈ lipid profile	[127]
250 mg OLE	pc, db, RCT	daily application (12 weeks)	30/group	hypertension participants age: 23.4 ± 1.4 years BMI: 22.7 ± 3.0	≈ fasting plasma glucose, insulin ≈ liver enzymes ↓ inflammatory cytokines (TNF-α, IL-8, IL-6)	[125]
oleuropein (20 mg)	pc, db, RCT, co	single application	placebo: 20 verum: 20	healthy participants age: 33.9 ± 6.9 BMI: 20.7 ± 3.7	↓ postprandial plasma glucose ↑ postprandial plasma insulin ↓ postprandial oxidative stress ↑ GLP-1, ↓ DPP-4	[121]
20 mL OLE (136.2 mg oleuropein; 6.4 mg hydroxytyrosol)	pc, db, RCT, co	daily application (6 weeks)	placebo: 60 verum: 60	PHT participants age: 45.3 ± 12.7 years BMI: 27.0 ± 3.4	↓ SBP, DBP (slight reduction) ↓ total CH, LDL-C, TG, IL-8 ≈ vascular function, CRP, adiponectin ≈ fasting glucose, insulin, HOMA-IR, QUICKI, HDL-C	[126]
250 mg OLE (oleuropein ≥ 100 mg)	pc, db, RCT	daily application (12 month)	placebo: 32 verum: 32	OST participants verum/placebo: age: 59.72/59.35 years BMI: 25.90/27.52	↓ total CH, LDL-C, TG ≈ HDL-C	[129]
OLE (51.1 mg oleuropein; 9.7 mg hydroxytyrosol)	pc, db, RCT, co	daily application (12 weeks)	placebo: 46 verum: 46	overweight participants age: 46.4 ± 5.5 years BMI: 28.0 ± 2.0	↓ postprandial plasma glucose, insulin ↑ insulin sensitivity (Matsuda index) ↑ pancreatic β-cell function (disposition index) ≈ lipid profile, body fat proportion, ABP	[122]

RCTs are listed according to the publication date, whereby 6 RCTs sorted for highest cohort size (*n*) of available studies are given. Only main metabolic and cardiovascular endpoint measures are presented. pc: placebo-controlled, db: double-blind, co: crossover design, RCT: randomized clinical trial, PHT: pre-hypertensive, OST: osteoporosis, SBP: systolic blood pressure, DBP: diastolic blood pressure, CH: total cholesterol, LDL: low-density lipoprotein, HDL: high-density lipoprotein, TGs: triglycerides, HOMA-IR: homoeostasis model assessment-estimated insulin resistance, QUICKI: quantitative insulin sensitivity check index, OLE: olive leaf extract.

**Table 2:** Characteristics and main study outcomes of placebo-controlled, randomized, clinical trials investigating the effects of *Withania somnifera* on metabolic parameters.

Study Medication	Study Type	Dose Regimen	Cohort Size (*n*)	Study Cohort Criteria	Main Study Results Verum vs. Control (Increased: ↑; Decreased:↓; Unaffected: ≈)	Ref.
600 mg WSRE	pc, db, RCT	daily application (8 weeks)	placebo: 25 verum: 25	healthy athletes age: 18–≤45 years	↑ cardiorespiratory endurance: (↑ VO_2_ max outcome; ↑ TQR score; improved RESTQ score) ↑ anti-oxidative capacity	[153]
600 mg WSRE	pc, db, RCT	daily application (8 weeks)	placebo: 25 verum: 25	subclinical hypothyroid participants verum/placebo: age: 35.6/35.1 years	↑ T3, T4 ↓ TSH	[154]
600 mg WSRE (5% withanolides)	pc, db, RCT	daily application (8 weeks)	placebo: 25 verum: 25	chronic stressed, overweight participants	↓ perceived Stress Scale Score ↓ Food Cravings Questionnaire scores ↑ Oxford Happiness Questionnaire scores ↓ serum cortisol level	[151]
600 mg WSRE (5% withanolides)	pc, db, RCT	daily application (8 weeks)	placebo: 25 verum: 25	healthy participants undergoing resistance training verum/placebo: age: 28 ± 8/29 ± 9 years	↑ muscle strength, muscle size (upper body) ↓ body fat percentage	[152]

RCT, listed according to publication date are given. Only main metabolic and cardiovascular endpoint measures are presented. pc: placebo-controlled, db: double-blind, co: crossover design, RCT: randomized clinical trial, T3: triiodothyronine, T4: thyroxine, TSH: thyroid stimulating hormone, WSRE: withania somnifera root extract.

**Table 3:** Characteristics and main study outcomes of randomized clinical trials investigating the effects of *Vitis vinifera* on metabolic parameters.

Study Medication	Study Type	Dose Regimen	Cohort Size (*n*)	Study Cohort Criteria	Main Study Results Verum vs. Control (Increased: ↑; Decreased: ↓; Unaffected: ≈)	Ref.
300 mg GSE	pc, db, RCT	daily application (16 weeks)	placebo: 38 verum:40	mild hypertension participants verum/placebo: age: 56.4/56.9 years BMI: 25.2/26.1	↓ SBP, DBP (only in male participants) ↓ perceived stress score (PSQ)	[200]
200 mg GSE	pc, db, RCT, co	daily application (8 weeks)	placebo: 45 verum: 45	mild hyperlipidemia participants age: 48.22 ± 9.07 years	↓ CH, LDL, ox-LDL	[193]
200 mg GSE	pc, db, RCT	daily application (8 weeks)	placebo: 35 verum: 35	hyperlipidemia participants verum/placebo age: 46.6/47.3 years	↑ ApoA1, HDL ↑ PON activity ↓ CH, TG, LDL	[192]
300 mg GSE	pc, db, RCT	daily application (8 weeks)	placebo: 35 verum: 34	pre- and stage-I hypertension participants verum/placebo: age: 62.9/64.5 years BMI: 25.3/25.7	≈ SBP, DBP ≈ vasoactive markers	[204]
600 mg GSE	pc, db, RCT, co	daily application (4 weeks)	placebo: 32 verum: 32	T2D participants age: 61.8 ± 6.4 years BMI: 30.2 ± 5.9	↓ fructosamine, CH, CRP ↑ GSH ≈ fasting glucose, HOMA-IR ≈ endothelial function	[189]
900 mg GSE	pc, db, RCT, co	daily application (3 days)	placebo: 51 verum: 51	healthy participants age: 48.7 ± 14.3 years BMI: 25.6 ± 2.6	↓ 24 h energy intake (only in subjects with ≥7.5 MJ/day)	[197]

RCTs are listed according to publication date, whereby 6 RCTs for each group, sorted for highest cohort size (*n*) of available studies, are provided. Only main metabolic and cardiovascular endpoint measures are presented. pc: placebo-controlled, db: double-blind, co: crossover design, RCT: randomized clinical trial, SBP: systolic blood pressure, DBP: diastolic blood pressure, CH: total cholesterol, LDL: low-density lipoprotein, ApoA1: apolipoprotein A1, PON: paraoxonase, HOMA-IR: homoeostasis model assessment-estimated insulin resistance, CRP: c-reactive protein, GSH: reduced glutathione, GSE: grape seed extract.

**Table 4:** Characteristics and main study outcomes of randomized clinical trials investigating the effects of *Camellia sinensis* on metabolic parameters.

Study Medication	Study Type	Dose Regimen	Cohort Size (*n*)	Study Cohort Criteria	Main Study Results Verum vs. Control (Increased: ↑; Decreased: ↓; Unaffected: ≈)	Ref.
1500 mg GTE (856.8 mg EGCG)	pc, db, RCT, co	daily application (6 weeks)	placebo: 73 verum: 73	overweight participants age: 18–65 years BMI: ≥27	↓ LDL-C ↑ Leptin ≈ CH, TG, HDL	[247]
500 mg EGCG	pc, db, RCT	daily application (until birth)	placebo: 176 verum: 150	GDM participants verum/placebo: age: 29.6/28.7 years BMI: 25.9/26.2	↓ fasting plasma glucose and insulin ↓ HOMA-IR/HOMA-ß scores ↑ QUICK-I index	[238]
green tea/GTE/EGCG) (200 mg EGCG each)	pc, RCT, co	single application	placebo: 50 verum: 50	healthy participants age: 33.9 ± 7.6 years BMI: 23.7 ± 2.5	↑ FMD (only in the green tea group) ≈ NMD	[256]
GTE (843 mg EGCG; decaffeinated)	pc, db, RCT	daily application (12 month)	placebo: 473 verum: 463	healthy participants verum/placebo: age: 60.02/59.65 years BMI: 25.16/25.01	↓ CH, LDL ↑ TG (mainly obese, statin users)	[248]
1500 mg GTE (856.8 mg EGCG)	pc, db, RCT	daily application (12 weeks)	placebo: 38 verum: 39	obese participants verum/placebo: age: 44.1/44.9 years BMI: 31/30	↓ CH, LDL	[246]
1060 mg GTE (431.5 mg EGCG)	pc, db, RCT, co	daily application (6 weeks)	placebo: 65 verum: 63	obese participants verum/placebo: age: 49.5/49.4 years BMI: 31.7/31.4	≈ blood pressure ≈ body weight (only slight reduction during intervention period 1)	[251]

RCTs are listed according to the publication date, whereby 6 RCTs for each group, sorted for highest cohort size (*n*) in available studies, are provided. Only the main metabolic and cardiovascular endpoint measures are presented. pc: placebo-controlled, db: double-blind, co: crossover design, RCT: randomized clinical trial, GDM: gestational diabetes mellitus, CH: total cholesterol, LDL: low-density lipoprotein, TGs: triglycerides, HOMA-IR: homoeostasis model assessment-estimated insulin resistance, QUICKI: quantitative insulin sensitivity check index, FMD: flow-mediated dilation, NMD: nitro-mediated dilation, GTE: green tea extract, EGCG: epigallocatechin 3-gallate.

**Table 5:** Summary of main study outcomes of double-blind, placebo-controlled RCTs evaluating the effects of *Olea europea, Withania somnifera, Vitis vinifera*, and *Camellia sinensis* phytochemicals on metabolic syndrome parameters.

MetS Parameter	*Olea europea*	*Withania somnifera*	*Vitis vinifera*	*Camellia sinensis*
glucose metabolism	improvement of postprandial plasma glucose	only preclinical data available	no clear impact	improvement of glucose metabolism
dyslipidemia	improvement of single lipid parameters	only preclinical data available	improvement of single lipid parameters	improvement of single lipid parameters
cardiovascular alterations	improvement of vascular function	improved cardiorespiratory endurance	improvement of blood pressure parameters	improvement of cardiovascular parameters
	no clear impact on blood pressure			
obesity/ weight management	no clear impact	reduced perceived stress; reduced food craving; enhanced body weight reduction during reistance training	enhanced body weight reduction during caloric restriction	no clear impact
NAFLD	only preclinical data available	only preclinical data available	only preclinical data available	only preclinical data available
RCT quantity	*n* = 11	*n* = 4	*n* = 21	*n* = 28

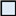
 No RCTs or only 1 RCT available for this parameter. 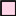 <50% of available RCTs show the effectivity of study medication on the respective parameter. 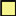 =50% of available RCTs show the effectivity of study medication on the respective parameter. 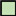 ≥50% of available RCTs show the effectivity of study medication on the respective parameter.

## Data Availability

Not applicable.
